# Customized SAM-Med3D with multi-view adapter and T2-FLAIR mismatch features for glioma IDH genotyping and grading

**DOI:** 10.3389/fnbeh.2025.1705385

**Published:** 2026-01-12

**Authors:** Xinyu Li, Hui Li, Yunyi Hu, Jingjing Zhang, Lanlan Wang, Xinran Yang

**Affiliations:** 1 School of Computer Science and Engineering, Central South University, Changsha, China; 2 School of Informatics, Xiamen University, Xiamen, China; 3 School of Information Resource Management, Renmin University of China, Beijing, China

**Keywords:** deep learning, glioma, grading, IDH genotyping, medical foundational model, multi-modal MRIs

## Abstract

**Objective:**

Gliomas, the most aggressive type of brain tumor, are infamous for their low survival rates. Tumor grading and isocitrate dehydrogenase (IDH) status are key prognostic biomarkers for gliomas. However, obtaining these markers typically requires invasive methods such as biopsy. As an effective, noninvasive alternative, multimodal MRI can reveal tumor spatial information and the microenvironment. Low-grade and IDH-mutant gliomas often exhibit T2-FLAIR mismatch signals. Medical image foundational models can explore complex representations in medical images, and fine-tuning them may further enhance glioma diagnosis.

**Methods:**

We propose a multi-task network, MTSAM, for simultaneous glioma IDH genotyping and grading. MTSAM first uses dilated convolutions to simulate large-field convolutions and then reviews the T2 and FLAIR images. Then, we employ convolutions to perform a detailed exploration of the T2 and FLAIR images, and we subtract the weighted T2 and FLAIR images to obtain T2-FLAIR mismatch features. T2-FLAIR mismatch features are concatenated with multimodal MRIs and input into the customized SAM-Med3D. The customized SAM-Med3D is fine-tuned by leveraging complementary information across multi-view modalities, including MRIs, handcrafted radiomics (HCR), and clinical features. Then it extracts deep features for accurate IDH genotyping and grading.

**Results:**

MTSAM achieves AUCs of 92.38 and 94.31% for glioma IDH typing and grading on the UCSF-PDGM dataset, respectively, and AUCs of 91.56 and 93.37% on the BraTS2020 dataset, outperforming other methods. Additionally, we use Grad-CAM to visualize the attention maps of MTSAM, demonstrating its potential for non-invasive glioma diagnosis.

**Conclusion:**

The proposed method demonstrates that we can effectively fuse multi-view, non-invasive information and fully explore the knowledge learned by medical image foundational models from large-scale medical datasets to facilitate glioma diagnosis, thereby advancing glioma research.

## 1 Introduction

Glioma is the most common malignant primary brain tumor in adults and has gained notoriety for its extremely poor five-year survival rate (Weller et al., 2015; Jayaram and Phillips, 2024; Li et al., 2022a). In clinical practice, the formulation of preoperative treatment plans for gliomas, such as whether to perform total surgical resection or whether preoperative targeted therapy is needed, depends heavily on tumor grading and isocitrate dehydrogenase (IDH) status (Olar et al., 2015; van den Bent et al., 2024). According to the World Health Organization (WHO) Classification of Tumors of the Central Nervous System (Mahajan et al., 2022; Wen and Packer, 2021), adult diffuse gliomas are classified into Grades 2–4, with IDH wild-type glioblastoma having the highest annual incidence. Specifically, the prognosis of IDH-mutant gliomas is generally better than that of IDH-wildtype gliomas (Vettermann et al., 2019; Han et al., 2020). High-grade gliomas are highly malignant and invasive, leading to a poor prognosis (Navarria et al., 2022; Zhou et al., 2022), while low-grade gliomas are less malignant and less invasive, resulting in a relatively better prognosis (Khan et al., 2021). Therefore, more conservative treatment approaches should be adopted for patients with IDH-wildtype or high-grade gliomas. Traditional methods for evaluating IDH status rely on invasive tissue sampling (Yu et al., 2024; Lim-Fat et al., 2022), which carries risks such as bleeding, infection, and tumor metastasis. For gliomas located in deep-seated areas such as the brainstem or thalamus, or with small volumes, the surgical risk of invasive biopsy is extremely high, and approximately 20% of such patients are forced to delay diagnosis because the benefits of biopsy are outweighed by the risks (Esquenazi et al., 2018; Santos et al., 2024). Moreover, gliomas often exhibit high spatial heterogeneity (Nicholson and Fine, 2021). Multimodal magnetic resonance imaging (MRI) and hand-crafted radiomics (HCR) features have emerged as promising approaches for glioma diagnosis due to the rich spatial information they contain (Tan et al., 2019; van Santwijk et al., 2022; Dayarathna et al., 2024; Yang et al., 2025; Bijari et al., 2025; Wu et al., 2023; Sudre et al., 2020). Clinical features such as age and gender provide fundamental physiological information about gliomas (Li et al., 2022b; Akpinar and Oduncuoglu, 2025). These non-invasive multi-view data offer crucial information for glioma diagnosis.

In IDH-wildtype gliomas, cells undergo metabolic reprogramming via the “Warburg effect,” which enables the rapid generation of adenosine triphosphate (ATP) and produces large quantities of biosynthetic precursors such as pyruvate and glutamine (Braun et al., 2021; Murnan et al., 2023). These substances provide raw materials for the synthesis of DNA and proteins required for the rapid division of tumor cells, thereby accelerating proliferation. IDH-wildtype gliomas generally present at higher grades, suggesting a potential complementary relationship between IDH status and glioma grade. Specifically, the incidence of IDH mutations is approximately 12% in WHO Grade 4 gliomas, while this proportion is nearly 60% in Grade 3 gliomas (Jusue-Torres et al., 2023; Komori, 2022; Whitfield and Huse, 2022). Using a multi-task deep learning network to simultaneously perform IDH genotyping and glioma grading may enable exploration of the potential complementary relationship between IDH status and grade, thereby further enhancing glioma diagnosis (Sairam et al., 2023).

The T2-FLAIR mismatch signal is critical for glioma IDH genotyping and grading (Han et al., 2022; Park et al., 2021; Lee et al., 2024). Specifically, IDH-mutant gliomas and lower-grade gliomas are often closely associated with this characteristic T2-FLAIR mismatch signal. However, traditional methods (Jeon et al., 2025; Tang et al., 2024) rely on T2-FLAIR image subtraction to explore T2-FLAIR mismatch features, treating all image positions as having uniform weights, and struggling to effectively capture the subtle differences and mismatch signals between T2 and FLAIR images. The perceptual logic of the human visual system (Stewart et al., 2020) suggests that forming an overall overview first, then conducting detailed observation, can effectively capture differential information. Therefore, first performing an overview of the T2 and FLAIR images, then conducting a refined exploration of their complementary information for weighted subtraction, may further reveal T2-FLAIR mismatch features.

Medical image foundational models are capable of mining complex patterns in data (Moor et al., 2023; Willemink et al., 2022), yet their potential for IDH genotyping and grading of gliomas has not been fully explored (Zhang and Metaxas, 2024; He et al., 2024). The encoder of segmentation models can effectively capture tumor edge and location information, which contains rich prognostic information (Zhang J. et al., 2023; Cheng et al., 2022; Yu et al., 2024). Thus, using features extracted by segmentation encoders may further improve the performance of IDH genotyping and glioma grading. SAM-Med3D (Wang et al., 2025) is a foundational model for medical image segmentation, pre-trained on a large-scale dataset comprising 245 disease categories, 70 public datasets, and 8,000 privately authorized hospital cases, including 22,000 medical images and 143,000 corresponding segmentation masks. Additionally, SAM-Med3D is composed of transformer blocks that focus on exploring long-range dependencies in images and achieves an overall Dice score of 80.71% on 16 medical image segmentation datasets. However, when SAM-Med3D is applied to medical image diagnosis, its performance often remains suboptimal (Wang, 2025). Fine-tuning SAM-Med3D by fusing multi-view information, including multimodal MRIs, HCR, and clinical features, can leverage the prior knowledge that SAM-Med3D has learned from large-scale medical datasets, thereby improving IDH genotyping and grading performance for gliomas.

To improve the performance of glioma IDH genotyping and grading, we propose the multi-task network named MTSAM. MTSAM uses the customized SAM-Med3D to simultaneously conduct IDH genotyping and grading for gliomas. First, we employ dilated convolutions and convolutions with shared weights to respectively conduct an overview and detailed exploration of the complementary information between T2 and FLAIR images. We then perform a weighted subtraction of these two images to obtain T2-FLAIR mismatch features. Then, we concatenate the T2-FLAIR mismatch feature maps with multi-modal MRIs along the channels and feed them into the customized SAM-Med3D, which is fine-tuned by fusing multi-view information, to obtain deep features for IDH genotyping and grading. We employ an uncertainty-weighted method to balance the losses associated with IDH genotyping and grading. Overall, our main contributions include the following:

(1) We propose a multi-task network named MTSAM, which explores T2-FLAIR mismatch features and utilizes the customized SAM-Med3D to explore the SAM-Med3D's prior knowledge learned from large-scale medical data for accurate glioma IDH genotyping and grading.

(2) We propose a multi-view adapter called MVAdapter that explores complementary and multi-scale information in multi-view data, including HCR, clinical, and MRI features, to fine-tune SAM-Med3D and uncover deep features for glioma IDH genotyping and grading.

(3) We propose a T2-FLAIR mismatch feature extraction block based on the human visual system, named MFEB, which first provides an overview of MRIs through dilated convolutions and then conducts detailed exploration using convolutions, aiming to capture the complementary information between T2 and FLAIR images and perform weighted subtraction to obtain T2-FLAIR mismatch features.

## 2 Materials and methods

### 2.1 Datasets

We use the publicly available UCSF-PDGM (Calabrese et al., 2022) and BraTS2020 (Menze et al., 2014) datasets. Each sample includes T1-weighted, T1-contrast-enhanced (T1CE), T2-weighted, and fluid-attenuated inversion recovery (FLAIR) images, along with their segmentation results, IDH status, and grading information. After excluding samples with missing segmentation, IDH subtype, or grading information in the UCSF-PDGM and BraTS2020 datasets, 492 and 128 patients' data remain, respectively. Regions of interest (ROIs) are manually adjusted by radiologists and verified by experts, covering key areas such as the enhancing tumor region, necrotic tumor region, and peritumoral abnormal areas. We determine the center of the annotated tumor ROI by calculating the midpoint along its depth, width, and height. Using this center as a reference, we extract a (128, 128, 128) patch from the MRI data to retain peritumoral information (Cheng et al., 2020), which serves as the 3D MRI input to the network.

We split the data into a training set and a test set in an 8:2 ratio. 15% of the training data is used for validation to fine-tune parameters. As shown in Table 1, the demographic distributions in the training set and the test set are consistent.

**Table 1:** Summary of the datasets used in this study.

	**UCSF-PDGM**		**BraTS2020**	
	**Training**	**Testing**	**Training**	**Testing**
Subject *n*	394	98	120	28
Male *n*	239 (0.6)	56 (0.6)	62 (0.5)	15 (0.5)
Age mean	57	57	52	51
**Grade, decile**				
Lower- Grade (2,3)	77 (0.2)	22 (0.2)	53 (0.4)	11 (0.4)
Higher- Grade (4)	317 (0.8)	76 (0.8)	67 (0.6)	17 (0.6)
**IDH status, decile**				
Wildtype	312 (0.8)	77 (0.8)	73 (0.6)	18 (0.6)
Mutant	82 (0.2)	21 (0.2)	47 (0.4)	10 (0.4)

### 2.2 Overview of MTSAM

**Figure 1:**
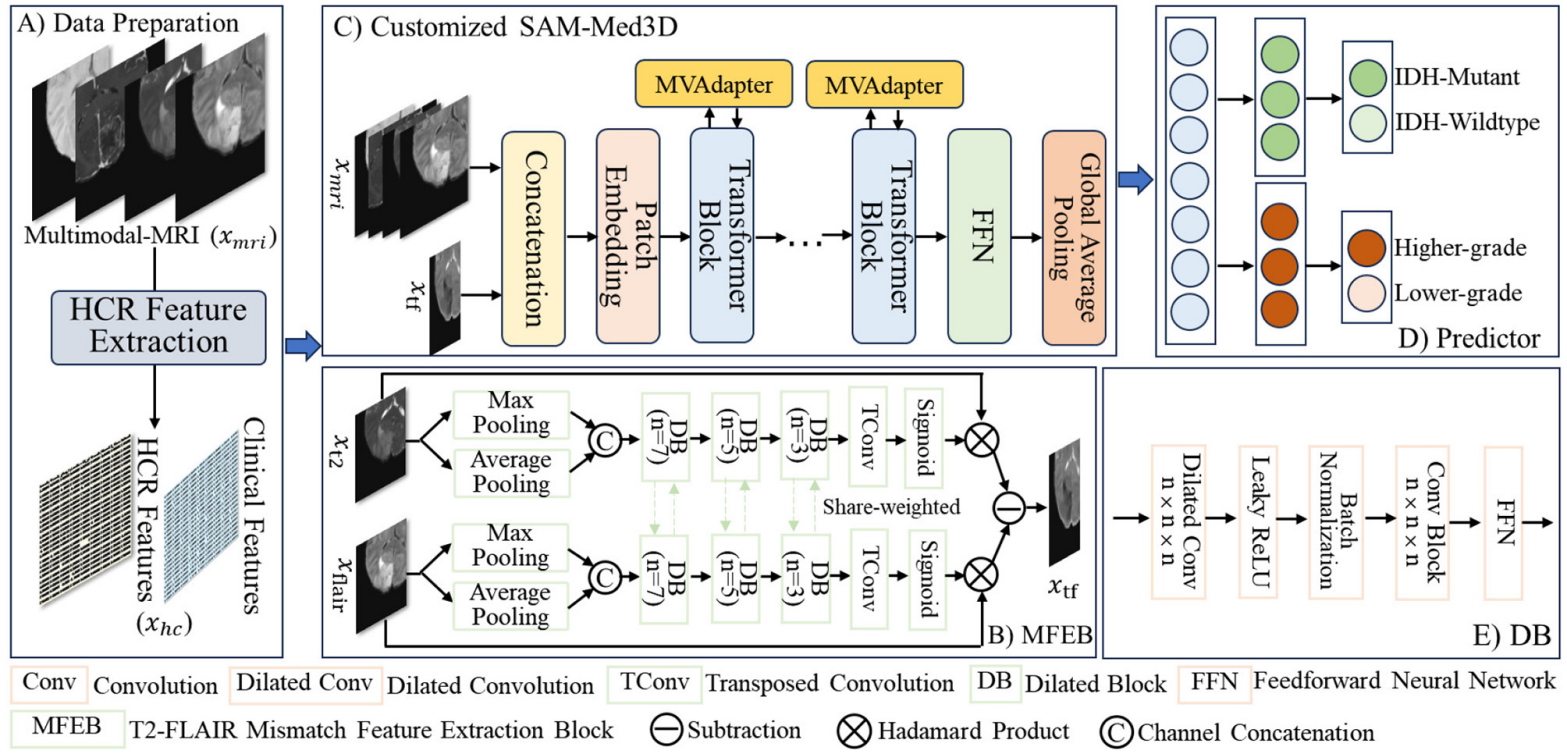
Overall pipeline of the proposed MTSAM. **(A)** Data preparation. **(B)** MFEB. **(C)** Customized SAM-Med3D. **(D)** Predictor. **(E)** DB.

As shown in Figure 1, the main steps of MTSAM are as follows: (A) Firstly, MTSAM takes multi-modal MRI as 3D input, extracts 1D hand-crafted radiomics features from the multi-modal MRI, and combines them with clinical features. (B) MTSAM mimics the human eye system, which uses multi-scale dilated blocks first to take an overview and then explore in detail to explore the complementary information between T2 and FLAIR images. After weighting them respectively, it extracts the T2-FLAIR mismatch feature map. (C) MTSAM concatenates the T2-FLAIR mismatch features and the multi-modal MRIs along the channels. The combined data is then input into the customized SAM-Med3D, fine-tuned by adapters that fuse multi-view information and further extract deep features for glioma IDH genotyping and grading.

#### 2.2.1 HCR and clinical feature extraction

As shown in Figure 1, MTSAM first extracts HCR features (Liu et al., 2019) from multi-modal MRI and their corresponding Regions of Interest (ROIs). The detailed extraction method for HCR features is available at https://pyradiomics.readthedocs.io/en/latest/. A total of 2,153 HCR features are extracted from each sample, and these features are combined with clinical features, including patients' age and gender, to form 1D features. The value of each feature is standardized by subtracting the mean and dividing by the standard deviation. In the UCSF-PDGM training dataset, we use Lasso regression with five-fold cross-validation to perform HCR feature selection for IDH genotyping and grading, respectively. Features with a *p*-value greater than 0.05 are considered redundant and thus removed. The 1D features selected in each fold for IDH genotyping and grading are combined. Ultimately, we select 99 HCR features, including 18 first-order, 21 gray level size zone matrix (glszm), 16 gray level dependence matrix (gldm), 19 gray level co-occurrence matrix (glcm), 10 neighboring gray tone difference matrix (ngtdm), 10 gray level run length matrix (glrlm), and five shape-based HCR features, as well as two clinical features, including age and gender. We use the 101 1D features selected from the UCSF-PDGM dataset for the BraTS2020 dataset to ensure that the HCR and clinical features used in the two datasets remain consistent.

#### 2.2.2 T2-FLAIR mismatch feature extraction

Mismatch information between T2 and FLAIR can assist in IDH genotyping and glioma grading (Han et al., 2022; Park et al., 2021; Lee et al., 2024). Subtracting T2 and FLAIR images directly will treat all regions equally and may ignore key mismatch information. Therefore, we propose the MFEB, which extracts complementary features from T2 and FLAIR images and performs weighted subtraction to explore T2-FLAIR mismatch features.

##### 2.2.2.1 Complementary feature extraction

We first explore the complementary information between T2 and FLAIR. We use global average pooling and max pooling, respectively, to comprehensively aggregate the local information from T2 and FLAIR images, which can be expressed as follows:


$$x_{m}^{p}=P_{avg}(x_{m})⊕P_{max}(x_{m}),\;m\in \{t2,\;flair\}),$$ (1)

where *P*_*avg*_ and *P*_*max*_ represent global average pooling and max pooling, respectively, which reduce the MRI resolution to half. *x*_*t*2_ and *x*_*flair*_ are the T2 and FLAIR images, respectively. ⊕ denotes channel concatenation. We then design multi-scale shared weight dilated blocks (DB) that first use dilated convolutions with a larger field of view to obtain an overview and then use convolution blocks to explore details to uncover the complementary information between T2 and FLAIR images, which can be expressed as:


$$\begin{array}{c} F_{db}^{n}\left(x_{m}^{p}\right)=FFN\left(f_{c}^{n}\left(drop\left(\beta \left(f_{c}^{n}\left(BN\left(\beta \left(f_{d}^{n}\left(x_{m}^{p}\right)\right)\right)\right)\right)\right)\right)\right), \end{array}$$ (2)

where $f_{d}^{n}$ represents the dilated convolutions with a kernel size of n and a dilation rate of 3, while $f_{c}^{n}$ represents convolutions with a kernel size of n. *drop* is the Dropout function. β is the Leaky ReLU activation function, *BN* refers to Batch Normalization, and *FFN* is a feed-forward neural network composed of two pointwise convolutions and a Leaky ReLU activation function.

##### 2.2.2.2 Weighted subtraction

We adopt multi-scale DBs with shared weights to explore the complementary information between T2 and FLAIR images and weight them respectively, which can be expressed as follows:


$$\begin{array}{c} x_{t2}^{w}=\rho \left(f_{tc}\left(F_{db}^{3}\left(F_{db}^{5}\left(\left(F_{db}^{7}\left(x_{t2}^{p}\right)\right)\right)\right)\right)\right)\times x_{t2}, \end{array}$$ (3)

$$\begin{array}{c} x_{flair}^{w}=\rho \left(f_{tc}\left(F_{db}^{3}\left(F_{db}^{5}\left(\left(F_{db}^{7}\left(x_{flair}^{p}\right)\right)\right)\right)\right)\right)\times x_{flair}, \end{array}$$ (4)

where *f*_*tc*_ represents transposed convolutions and ρ represents the sigmoid activation function. Finally, we subtract the weighted T2 and FLAIR images to obtain the T2-FLAIR mismatch features, which can be expressed as:


$$\begin{array}{c} x_{tf}=x_{t2}^{w}-x_{flair}^{w}. \end{array}$$ (5)

Next, the T2-FLAIR mismatch features *x*_*tf*_ are concatenated along the channels with the multi-modal MRIs *x*_*mri*_. The combined data are then input into the customized SAM-Med3D to extract deep features for glioma IDH genotyping and grading.

#### 2.2.3 Customized SAM-Med3D for feature extraction

To effectively leverage the prior knowledge that SAM-Med3D has learned from large-scale datasets, we fuse multi-view features and conduct comprehensive deep feature extraction to fine-tune SAM-Med3D for glioma diagnosis. The steps of the customized SAM-Med3D can be divided into multi-view feature fusion, deep feature extraction, and fine-tuning SAM-Med3D.

##### 2.2.3.1 Multi-view feature fusion

**Figure 2:**
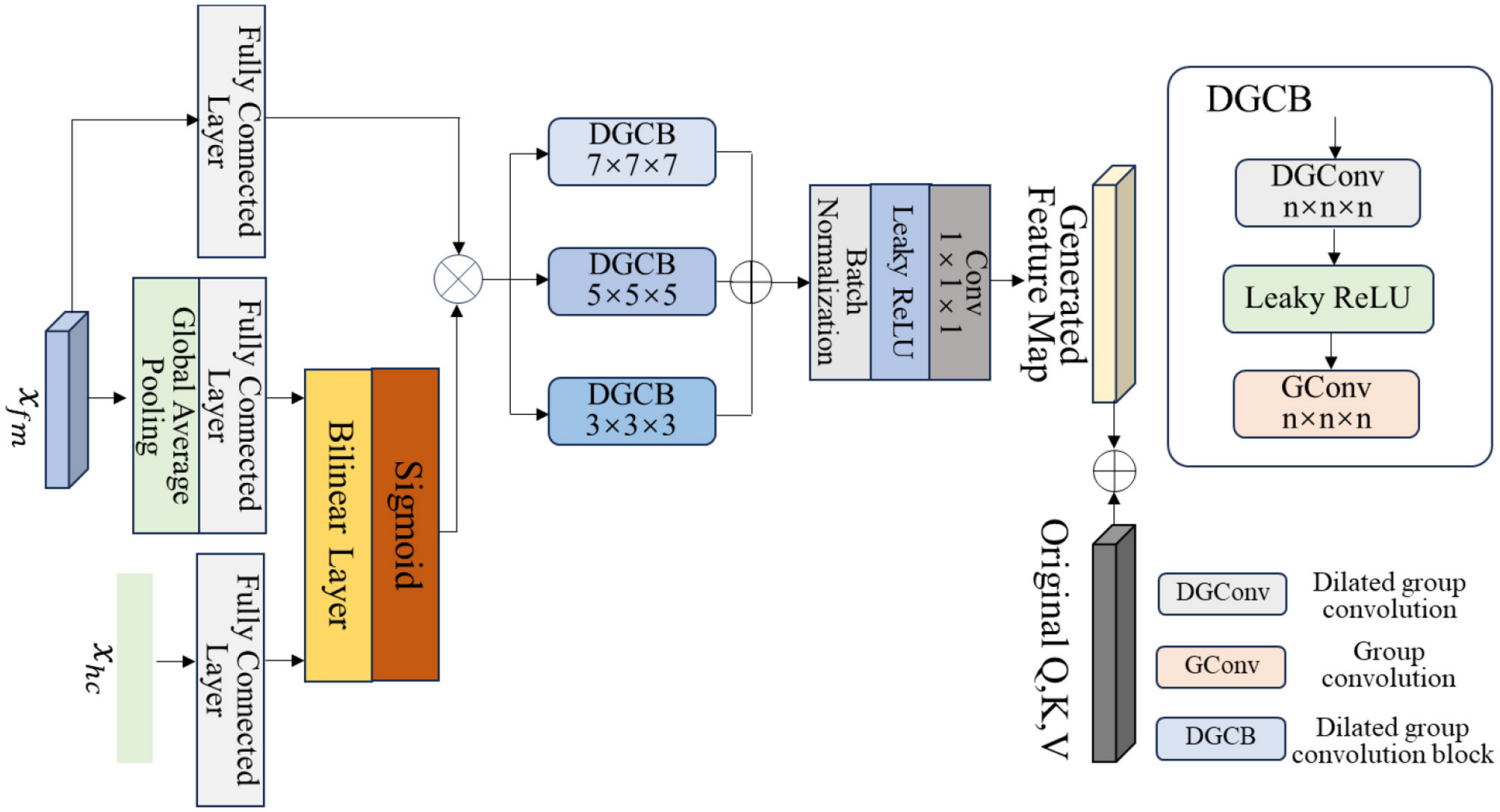
Details of multi-view adapter (MVAdapter).

We design MVAdapter to explore the complementary information among multi-view data to fine-tune the attention mechanism of SAM-Med3D for IDH genotyping and grading. As shown in Figure 1C, the fine-tuned SAM-Med3D encoder consists of a patch embedding layer and multiple transformer blocks. First, we expand the convolutional weights of the Patch Embedding layer fivefold to accommodate the concatenated *x*_*tf*_ and *x*_*mri*_. After passing through the Patch Embedding layer, we obtain the feature map *x*_*fm*_ with a shape of (8, 8, 8) and a channel number of 384. As shown in Figure 2, to effectively fine-tune SAM-Med3D, we first explore the complementary information among multi-view data, including 3D MRI images, 1D HCR, and clinical features. Specifically, we perform global average pooling along the channel dimension on the feature maps of MRI images to convert them into 1D features, then use a fully connected layer to reduce the number of channels to one-eighth, yielding 48 features. Similarly, we use another fully connected layer to reduce the 101 HCR and clinical features to 48 features. We use a bilinear layer to explore the complementary information among the multi-view, including MRIs, HCR, and clinical features, to weight the feature maps *x*_*fm*_, which can be expressed as follows:


$$\begin{array}{c} x_{fm}^{w}=\rho \left(f_{bil}\left(f_{fc}\left(P_{avg}^{c}\left(x_{fm}\right)\right),f_{fc}\left(x_{hc}\right)\right)\right)\times f_{fc}\left(x_{fm}\right), \end{array}$$ (6)

where *f*_*fc*_ represents the fully connected layers, *f*_*bil*_ represents the bilinear layers, and $P_{avg}^{c}$ represents global average pooling along the channels.

##### 2.2.3.2 Deep feature extraction

To fully explore the deep information in the multi-view fused feature $x_{fm}^{w}$, we design multi-scale dilated group convolution blocks (DGCB). Specifically, we utilize multi-scale dilated group convolutions and group convolutions to extract deep information from the weighted feature map $x_{f_{fm}^{w}}$. We then use pointwise convolution to explore inter-channel information and reduce the number of channels to 1,052 to obtain deep multi-view fused features, which is consistent with the dimensions of the query, key, and value generated by the transformer block. The process of deep feature extraction can be expressed as follows:


$$\begin{array}{l} x_{fusion}=f_{pc}(\beta ((BN(f_{gc}^{7}(\beta (f_{dgc}^{7}(x_{fm}^{w})))+f_{gc}^{5}(\beta (f_{dgc}^{5}(x_{fm}^{w})))\\ \;+f_{gc}^{3}(\beta (f_{dgc}^{3}(x_{fm}^{w})))))), \end{array}$$ (7)

where $f_{dgc}^{n}$ represents the dilated grouped convolutions with a kernel size of *n* and a dilation rate of 3. $f_{gc}^{n}$ denotes the grouped convolutions with a kernel size of *n*. *f*_*pc*_ is a pointwise convolution.

##### 2.2.3.3 Fine-tuning SAM-Med3D

We add the deep multi-view fused features to the generated query, key, and value in the transformer block to fine-tune SAM-Med3D for glioma diagnosis. The process of fine-tuning SAM-Med3D can be expressed as:


$$\begin{array}{c} F_{ft}\left(x_{fusion},x_{qkv}\right)=x_{fusion}+x_{qkv}, \end{array}$$ (8)

where *x*_*qkv*_ denotes the query, key, and value vectors produced by the transformer blocks in SAM-Med3D. Finally, the feature maps from the fine-tuned SAM-Med3D encoder are passed through global average pooling to extract deep features for both glioma IDH genotyping and grading.

### 2.3 Joint loss for IDH genotyping and grading

#### 2.3.1 Weighted cross-entropy loss

To optimize the trainable parameters of MTSAM, we use the prediction results from its IDH genotyping and grading as inputs to the loss function. To address data imbalance, we design a weighted cross-entropy loss function that assigns higher weights to samples with lower occurrence frequencies. Additionally, we increase the loss weight for samples that are difficult to classify, thereby reducing the influence of easily classified samples. The weighted cross-entropy loss function can be expressed as:


$$\begin{array}{c} L\left(y_{t}\right)=-y_{t}\log\left(y_{t}\right)-\left(1-y_{t}\right)\log\left(1-y_{t}\right), \end{array}$$ (9)

$$\begin{array}{c} L\left(y_{t}\right)=-\alpha {\left(1-y_{t}\right)}^{\gamma }\log\left(y_{t}\right)+\beta \cdot L\left(y_{t}\right), \end{array}$$ (10)

where *L*(*y*_*t*_) is the standard binary cross-entropy loss, and *y*_*t*_ is the predicted probability of the true glioma class. α is a scaling factor used to adjust the importance of classes according to the class imbalance. γ is the focusing parameter that controls the strength of the modulating factor ${\left(1-y_{t}\right)}^{\gamma }$. By reducing the loss for easily classified samples, they enable the model to focus on hard-to-classify samples. Based on empirical engineering, for the weighted cross-entropy loss function in glioma IDH genotyping and grading, α is set to the ratio of the different classes.γ is set to 2. β is the regularization factor for the cross-entropy term, used to balance the weighted modulating term and the original cross-entropy loss.

#### 2.3.2 Joint loss with uncertain loss weight

We adopt an uncertain-loss-weight approach Kendall et al. (2018) to jointly optimize the losses for IDH genotyping and grading using a weighted cross-entropy loss. The joint loss function can be expressed as:


$$\begin{array}{c} L_{\text{joint}}=\overset{2}{\underset{i=1}{\sum }}\frac{1}{2\gamma_{i}^{2}}L_{i}+\log\overset{2}{\underset{i=1}{\prod }}\gamma_{i}, \end{array}$$ (11)

where $L_{1}$ and $L_{2}$ correspond to the losses for glioma IDH genotyping and grading obtained via the weighted cross-entropy loss function, respectively. The γ_*i*_ is the weight parameter for each loss function, initially set to 1 based on empirical engineering and adjusted during training.

### 2.4 Implementation details

The training process is implemented using PyTorch, and experiments are conducted on NVIDIA 4090 GPUs. To enhance the generalization ability of model training, we apply random rotation, flipping, Gaussian noise, intensity transformation, and shifting to multi-modal MRIs. We use the Ranger (Wright and Demeure, 2021) optimizer with an initial learning rate of 2e-5, and the learning rate gradually decreases during training. The batch size is set to 4. The publicly available code and data for MTSAM can be found at https://github.com/mtsams/MTSAM.

### 2.5 Compared methods and evaluation metrics

To validate the superiority of MTSAM, we compare it with several methods for IDH genotyping and grading in gliomas. We ensure consistency across all compared methods and MTSAM in implementation details, including training settings, data augmentation strategies, and hardware environment. Specifically, regarding methods for glioma IDH genotyping, Tan et al. (2019) used a support vector machine to model HCR features. Yang et al. (2025) used Swin Transformer to extract deep features from MRI slices for IDH genotyping. Zhang H. et al. (2023) adopted a CNN+LSTM-based neural network to extract deep features from slices of multi-modal MRIs, respectively, and concatenated these deep features with HCR features for glioma IDH genotyping. Li et al. (2025) proposed DLRN, which uses MRI slices as input, extracts deep features using a fine-tuned pre-trained ResNet-101, and concatenates these features with HCR features for IDH genotyping using an SVM. Regarding methods for glioma grading, Qin et al. (2025) used an FFN to extract deep-level information from HCR features. Wu et al. (2023) proposed AGCN, which explores channel and spatial information in multi-modal MRI through a dual-domain attention mechanism and combines multi-scale features obtained using multi-branch convolution for glioma grading. Bijari et al. (2025) used three convolutional blocks to extract deep features from T1 images, which were then concatenated with HCR features for grading using logistic regression. Regarding methods for both glioma IDH genotyping and grading, Sudre et al. (2020) used a random forest with HCR and clinical features as inputs to perform these tasks separately. Sairam et al. (2023) used InceptionV3, with T1, T2, and FLAIR slices as inputs, to simultaneously perform glioma IDH genotyping and grading.

To evaluate the performance of the model, we use the area under the curve (AUC), accuracy (ACC), F1_score, and their 95% confidence intervals (CI) for the quantitative assessment of the model's IDH genotyping and grading, which can be represented as follows:


$$\begin{array}{c} ACC=\frac{TP+TN}{TP+TN+FP+FN}, \end{array}$$ (12)

$$\begin{array}{c} F1\text{\_}Score=2\times \frac{TP}{2TP+FP+FN}, \end{array}$$ (13)

where True Positives (TP) are the number of cases in which the model correctly predicts the positive class. True Negatives (TN) are the number of correctly predicted negative cases. False Positives (FP) are the number of incorrectly predicted positive cases, and False Negatives (FN) are the number of incorrectly predicted negative cases.

## 3 Results

### 3.1 Comparison with other methods

As shown in Table 2, MTSAM achieves AUCs of 92.38 and 94.31% for IDH genotyping and grading, respectively, on the UCSF-PDGM dataset, and AUCs of 91.56 and 93.37% on the BraTS2020 dataset, outperforming other methods. Specifically, compared with DLRN (Li et al., 2025), which uses multi-view information for IDH genotyping, MTSAM improves the AUC in IDH genotyping by 8.49 and 6.34%, respectively. Compared with the method of Bijari et al. (2025), which uses multi-view information for grading, MTSAM improves the AUC in grading by 9.62 and 5.07%, respectively. This may be attributed to MTSAM's effective exploration of the complementary information between IDH genotyping and grading. Compared with the multi-task method proposed by Sairam et al. (2023), MTSAM improves the AUC in IDH genotyping by 11.48 and 9.89%, respectively, and in grading by 9.63 and 8.2%, respectively, which could be because MTSAM effectively explores the prior knowledge that the medical foundational model has learned from large-scale medical data.

**Table 2:** Performance comparison with other methods.

**Method**	**Modality**	**IDH geno typing**			**Grading**		
		**AUC**	**ACC**	**F1_score**	**AUC**	**ACC**	**F1_score**
**UCSF-PDGM**							
Tang et al.	H	76.77 [69–84]	79.59 [72–86]	58.33 [44–72]	~	~	~
Yang et al.	M	83.45 [76–92]	79.59 [72–86]	62.96 [49–75]	~	~	~
Zhang et al.	M+H	82.84 [77–89]	80.61 [73–87]	66.67 [54–78]	~	~	~
DLRN	M+H	83.89 [78–87]	82.65 [77–89]	66.67 [52–78]	~	~	~
Qin et al.	H	~	~	~	80.24 [72–92]	80.61 [73–87]	87.74 [83–92]
AGCN	M	~	~	~	83.49 [73–93]	81.63 [76–88]	88.16 [83–93]
Bijari et al.	M+H	~	~	~	84.69 [73–93]	83.67 [78–90]	89.61 [85–94]
Sudre et al.	H	79.30 [73–85]	80.61 [73–87]	61.22 [46–75]	82.62 [72–92]	80.61 [74–87]	87.42 [82–92]
Sairam et al.	M	81.90 [77–86]	77.55 [70–85]	63.33 [50–75]	84.68 [75–91]	83.67 [78–90]	89.33 [85–93]
**MTSAM**	**M+H**	**92.38 [85–98]**	**90.82 [86–95]**	**80.85 [69–90]**	**94.31 [85–99]**	**92.86 [89–97]**	**95.36 [92–98]**
**BraTS2020**							
Tang et al.	H	76.67 [71–80]	75.00 [61–89]	69.57 [48–85]	~	~	~
Yang et al.	M	80.11 [75–85]	78.57 [64–89]	76.92 [62–86]	~	~	~
Zhang et al.	M+H	84.11 [79–90]	78.57 [66–87]	76.92 [61–88]	~	~	~
DLRN	M+H	85.22 [80–91]	82.14 [71–93]	80.00 [64–93]	~	~	~
Qin et al.	H	~	~	~	77.17 [73–99]	78.57 [68–89]	78.57 [63–91]
AGCN	M	~	~	~	83.17 [73–98]	82.14 [71–93]	82.76 [69–94]
Bijari et al.	M+H	~	~	~	88.30 [71–95]	85.71 [75–96]	86.67 [74–97]
Sudre et al.	H	85.56 [79–93]	71.43 [57–86]	71.43 [52–86]	80.89 [66–96]	75.00 [61–89]	74.07 [55–88]
Sairam et al.	M	81.67 [72–88]	78.57 [68–89]	76.92 [60–90]	85.17 [72–96]	82.14 [71–93]	82.76 [69–94]
**MTSAM**	**M+H**	**91.56 [85–97]**	**89.29 [79–96]**	**85.71 [70–97]**	**93.37 [79–100]**	**92.86 [82–100]**	**93.75 [85–100]**

M, multimodal MRIs; H, HCR and clinical features. Bold values indicate the highest performance among all compared methods.

### 3.2 Ablation study

#### 3.2.1 Effectiveness of T2-FLAIR mismatch features

To validate the effectiveness of T2-FLAIR mismatch features for glioma IDH genotyping and grading on the UCSF-PDGM dataset, we compared MTSAM with extracting T2-FLAIR mismatch features by direct subtraction without using MFEB (w/o MFEB) and the other that replaces the DB in MFEB with a convolutional layer (replace DB in MFEB). As shown in Table 3, replacing DB in MFEB with convolution reduces the ACC for both glioma IDH genotyping and grading by 1.02%. This may be because dilated convolutions can more effectively capture tumor information by simulating large-kernel convolutions, which is consistent with the fact that large-field-of-view convolutions facilitate glioma diagnosis (Wu et al., 2023; Zhu et al., 2025), thereby better capturing complementary information in T2 and FLAIR images. Additionally, without using MFEB to extract T2-FLAIR mismatch features, the AUCs for IDH genotyping and grading decrease by 2.09 and 1.73%, respectively, thereby validating the effectiveness of MFEB in extracting T2-FLAIR mismatch features.

**Table 3:** Ablation study of MTSAM.

**Method**	**IDH genotyping**			**Grading**		
	**AUC**	**ACC**	**F1_score**	**AUC**	**ACC**	**F1_score**
w/o MFEB	90.29 [78–99]	88.78 [83–94]	77.55 [65–87]	92.58 [85–98]	90.82 [86–95]	93.96 [90–97]
replace DB in MFEB	92.26 [85–99]	89.80 [85–94]	79.17 [67–88]	92.21 [84–99]	91.84 [87–96]	94.74 [91–98]
w/o H in MVAdapter	88.98 [80–98]	86.73 [81–92]	71.11 [56–82]	86.33 [78–94]	85.71 [80–92]	91.03 [87–95]
w/o M in MVAdapter	89.97 [85–96]	84.69 [79–91]	69.39 [56–81]	89.63 [83–95]	85.71 [80–92]	91.25 [87–95]
w/o MVAdapter	73.44 [67–78]	75.51 [68–83]	61.29 [48–72]	66.67 [59–80]	71.43 [57–86]	78.79 [72–85]
w/o IDH genotyping	~	~	~	93.51 [86–98]	91.84 [87–96]	94.74 [91–98]
w/o Grading	92.19 [85–98]	88.78 [84–94]	74.42 [60–86]	~	~	~
**MTSAM**	**92.38 [85–98]**	**90.82 [86–95]**	**80.85 [69–90]**	**94.31 [85–99]**	**92.86 [89–97]**	**95.36 [92–98]**

Bold values indicate the highest performance among all compared methods.

#### 3.2.2 Effectiveness of customized SAM-Med3D

To validate the effectiveness of fine-tuning SAM-Med3D by fusing multi-view features to extract deep features for IDH genotyping and grading, we compare MTSAM with the methods on the UCSF-PDGM dataset without using MVAdapter to fine-tune SAM-Med3D (w/o MVAdapter), without using HCR and clinical features in MVAdapter to fine-tune SAM-Med3D (w/o H in MVAdapter), and without using MRI features in MVAdapter to fine-tune SAM-Med3D (w/o M in MVAdapter). As shown in Table 3, without using MVAdapter to fine-tune SAM-Med3D for extracting deep features, the AUC of IDH genotyping and grading decreases by 18.94 and 27.64%, respectively, indicating that customized SAM-Med3D effectively exploits the prior knowledge learned by foundational medical image models from large-scale medical data.

#### 3.2.3 Effectiveness of multi-task learning

To validate the effectiveness of performing IDH genotyping and grading simultaneously, we compare MTSAM with methods that do not perform IDH genotyping (w/o IDH genotyping) or grading (w/o grading). As shown in Table 3, when IDH genotyping and grading are performed simultaneously, MTSAM achieves the best performance, validating that MTSAM can effectively exploit the complementary information across tasks.

### 3.3 Visualization analysis of MTSAM

**Figure 3:**
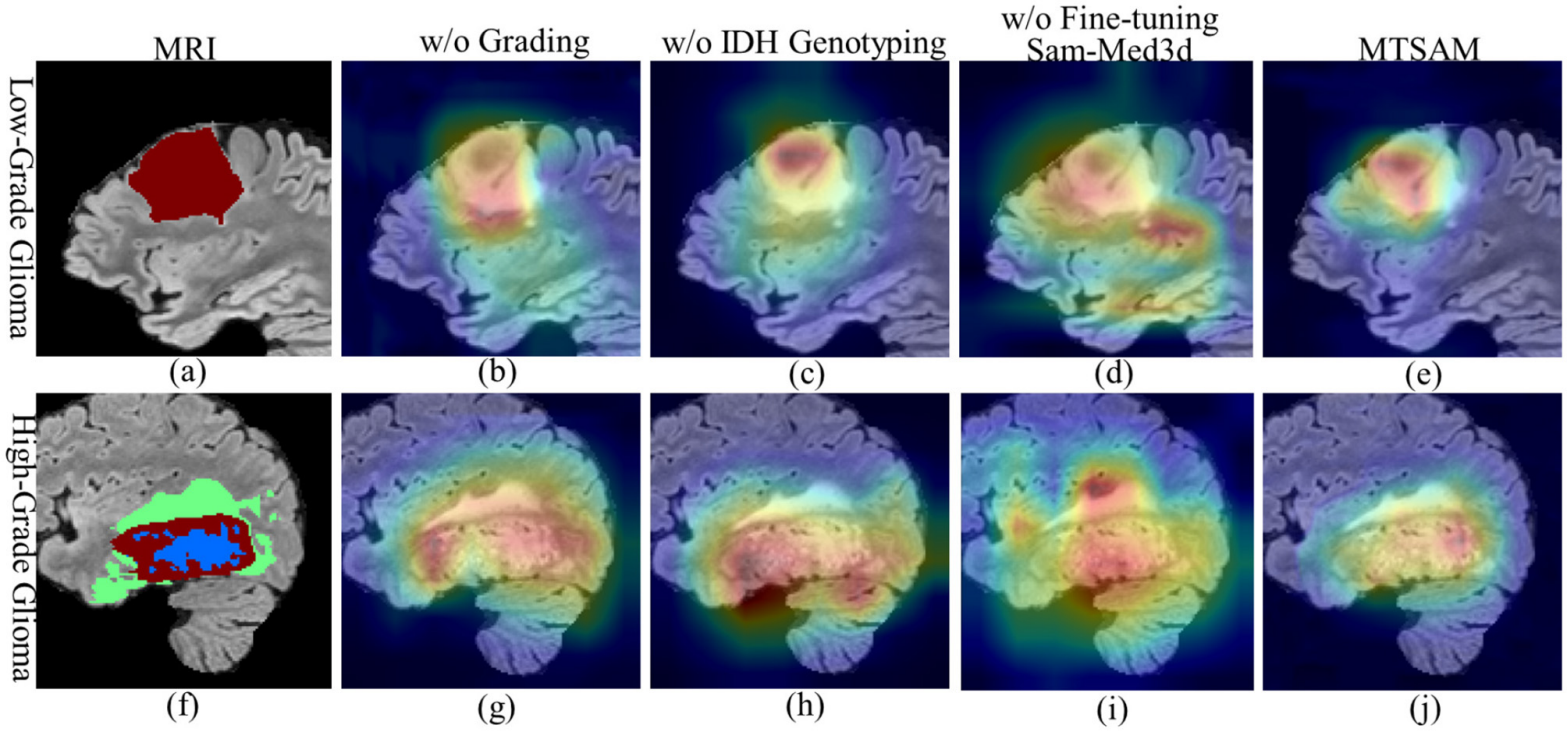
The visualization of attention maps in MTSAM. In MRIs, the blue, red, and green regions correspond to the necrotic tumor cores, enhancing tumors, and edema regions of gliomas, respectively.

To analyze the attention maps generated by MTSAM, we employ Grad-CAM (Selvaraju et al., 2020) to visualize the attention maps for non-fine-tuned and fine-tuned SAM-Med3D, without conducting IDH genotyping or grading. To ensure a fair comparison, the attention values across all visualization results were constrained to the same range, with red regions representing higher attention values and blue regions representing lower ones. As shown in Figures 3a, f, the blue, red, and green regions correspond to the necrotic tumor cores, enhancing tumors, and edema regions of gliomas, respectively. As shown in Figures 3b, c, g, h, when IDH genotyping or grading of gliomas is performed in isolation, the network tends to focus on non-tumor regions, underscoring the effectiveness of simultaneously performing both tasks. According to the WHO Classification of Tumors of the Central Nervous System (Wen and Packer, 2021; Horbinski et al., 2022), tumor necrotic regions are key indicators for glioma diagnosis. Specifically, low-grade gliomas have smaller tumor necrotic regions due to their low proliferation rate and sufficient blood supply. In contrast, patients with high-grade gliomas have larger tumor necrotic regions. Furthermore, highly invasive tumor cells are often present around the necrotic foci of gliomas, which is a key factor in determining the prognosis of gliomas (Ratliff et al., 2023; Markwell et al., 2022). As shown in Figures 3d, e, i, j, MTSAM with fine-tuned SAM-Med3D enables the network's attention regions to be more focused, with attention mainly concentrated in the necrotic tumor core regions, which contain rich prognostic information (Greenwald et al., 2024; Markwell et al., 2022; Liu et al., 2025), and highlighting the effectiveness of MTSAM in glioma diagnosis.

### 3.4 Visual of MTSAM with other methods

**Figure 4:**
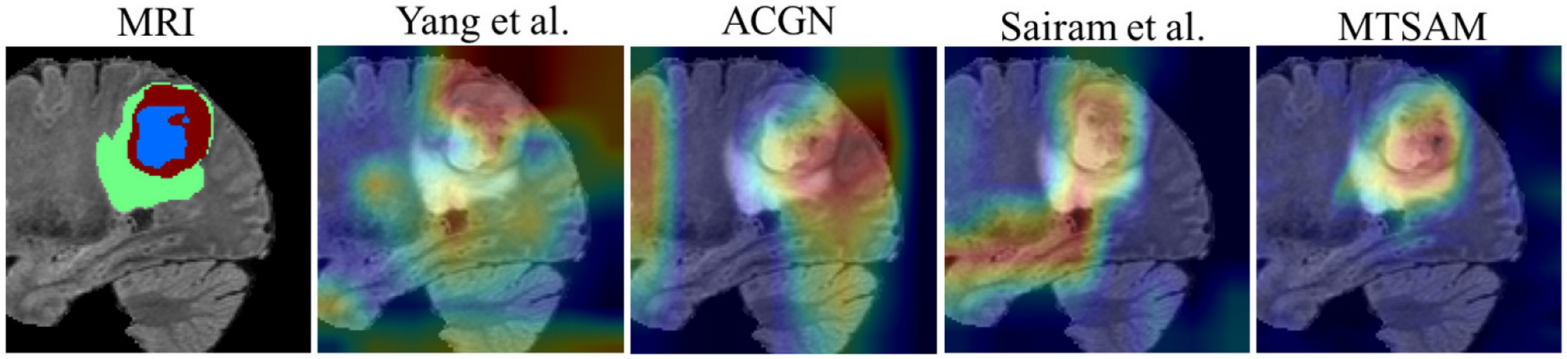
The visualization of different methods.

To further verify the effectiveness of MTSAM, we visualize MTSAM's attention map and compare it with those of other methods, including Yang et al. (2025), ACGN (Wu et al., 2023), and Sairam et al. (2023). As shown in Figures 4, we can observe that the attention of other methods is very scattered, while MTSAM's attention is concentrated on tumor regions, primarily the necrotic regions of the tumor. This may be because MTSAM effectively leverages the prior knowledge that the foundational model SAM-Med3D has learned from large-scale data.

### 3.5 Robustness validation of MTSAM

To validate the effectiveness of MTSAM, we conduct external validation. We collect 143 samples from TCGA-LGG (Pedano et al., 2016) and TCGA-GBM (Scarpace et al., 2016) that include glioma segmentation, IDH status, and grade information to perform external validation on the MTSAM as well as other multi-task methods, including Sairam et al. (2023) and Sudre et al. (2020) trained on the UCSF-PDGM dataset. As shown in Table 4, we find that MTSAM achieves an AUC of 83.28 and 80.33% for IDH typing and grading, respectively, on the TCGA dataset, outperforming other methods and validating the robustness of MTSAM.

**Table 4:** Validation of MTSAM on the TCGA Dataset.

**Method**	**IDH genotyping**			**Grading**		
	**AUC**	**ACC**	**F1_score**	**AUC**	**ACC**	**F1_score**
Sudre et al.	74.64 [79–90]	69.23 [62–76]	33.33 [20–45]	75.06 [68–82]	69.93 [64–76]	77.25 [71–82]
Sairam et al.	80.85 [75–88]	74.13 [68–80]	53.16 [41–64]	78.25 [74–87]	69.93 [63–76]	78.82 [73–84]
**MTSAM**	**83.28 [77–90]**	**81.82 [77–87]**	**71.11 [62–79]**	**80.33 [74–85]**	**74.13 [68–80]**	**80.42 [75–85]**

Bold values indicate the highest performance among all compared methods.

### 3.6 Effectiveness of MFEB

**Figure 5:**
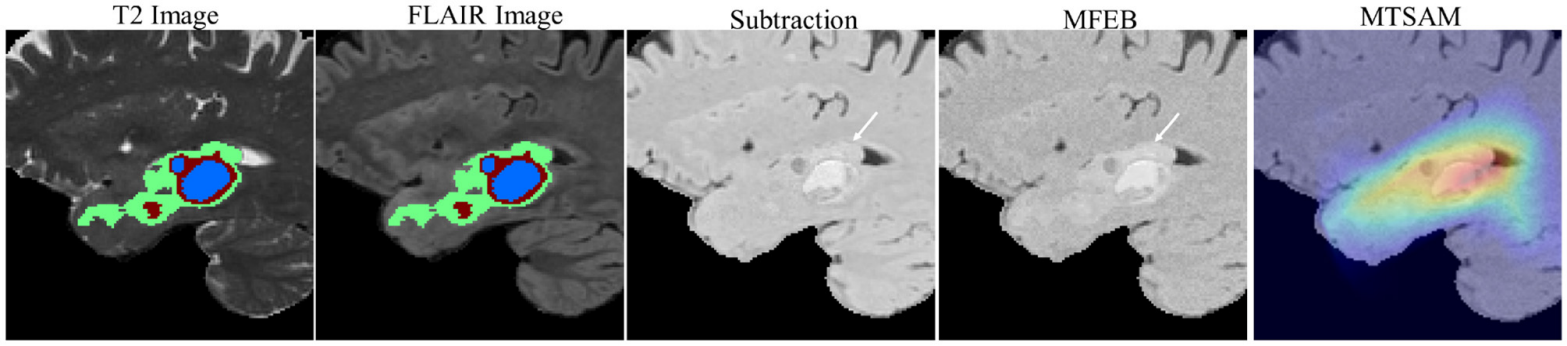
Visualization of T2-FLAIR mismatch feature.

To validate the effectiveness of MFEB, we visualize the direct subtraction results of T2 and FLAIR images, the T2-FLAIR mismatch features obtained via MFEB, and the attention maps of MTSAM on the MFEB-derived T2-FLAIR mismatch features. As shown in Figure 5, we observe that the T2-FLAIR mismatch features obtained via MFEB exhibit higher feature values near the tumor than the direct subtraction results. Additionally, the regions MTSAM focuses on in the T2-FLAIR mismatch feature maps are also around the tumor. This result demonstrates the potential of MFEB in exploring deep representations and validates its effectiveness in extracting T2-FLAIR mismatch features.

### 3.7 Selection of foundational model

To validate the effectiveness of using the medical foundational model SAM-Med3D for feature extraction in IDH genotyping and grading, we compare it with other medical foundational models on the UCSF-PDGM dataset. To ensure a fair comparison, all the foundational models, including SAM-Med2D (Cheng et al., 2023) and FastSAM3d (Shen et al., 2024), are fine-tuned using MVAdapter. As shown in Table 5, when using the fine-tuned FastSAM3D, derived from SAM-Med3D via knowledge distillation, for glioma IDH genotyping and grading, the AUC decreases by 0.63 and 0.56%, respectively. This may be because SAM-Med3D has learned effective prior knowledge from the large-scale medical data.

**Table 5:** Comparison with different foundational models.

**Method**	**IDH genotyping**			**Grading**		
	**AUC**	**ACC**	**F1_score**	**AUC**	**ACC**	**F1_score**
SAM-Med2D	84.23 [77–91]	83.67 [78–90]	60.00 [43–73]	80.22 [67–89]	79.59 [72–86]	86.84 [81–91]
FastSAM3D	91.75 [84–97]	87.76 [82–93]	76.00 [63–86]	93.75 [85–99]	91.84 [87–96]	94.59 [91–97]
**MTSAM**	**92.38 [85–98]**	**90.82 [86–95]**	**80.85 [69–90]**	**94.31 [85–99]**	**92.86 [89–97]**	**95.36 [92–98]**

Bold values indicate the highest performance among all compared methods.

### 3.8 Comparison with other fine-tuning methods

To validate the effectiveness of fine-tuning SAM-Med3D using MVAdapter, we compare it with other fine-tuning methods, including Bitfit (Ben Zaken et al., 2021), LoRA (Hu et al., 2022), and Side-tuning (Zhang et al., 2020), on the UCSF-PDGM dataset. As shown in Table 6, when SAM-Med3D is fine-tuned using side-tuning, the AUC in IDH genotyping and grading decreases by 4.81 and 9.82%, respectively. This may be because MVAdapter effectively exploits the complementary information among multi-view features to fine-tune SAM-Med3D and explore deep features.

**Table 6:** Comparison with different fine-tuning methods.

**Method**	**IDH genotyping**			**Grading**		
	**AUC**	**ACC**	**F1_score**	**AUC**	**ACC**	**F1_score**
Bitfit	80.77 [87–96]	77.55 [70–84]	64.52 [52–75]	78.20 [71–90]	75.51 [68–83]	83.33 [77–88]
LoRA	84.57 [76–91]	84.69 [79–91]	57.14 [40–73]	85.67 [73–93]	82.65 [77–89]	89.17 [85–93]
Side–tuning	87.57 [79–95]	85.71 [80–92]	68.18 [54–81]	84.49 [75–92]	84.69 [79–91]	90.45 [86–94]
**MVadapter**	**92.38 [85–98]**	**90.82 [86–95]**	**80.85 [69–90]**	**94.31 [85–99]**	**92.86 [89–97]**	**95.36 [92–98]**

### 3.9 Feature interpretability of MTSAM

**Figure 6:**
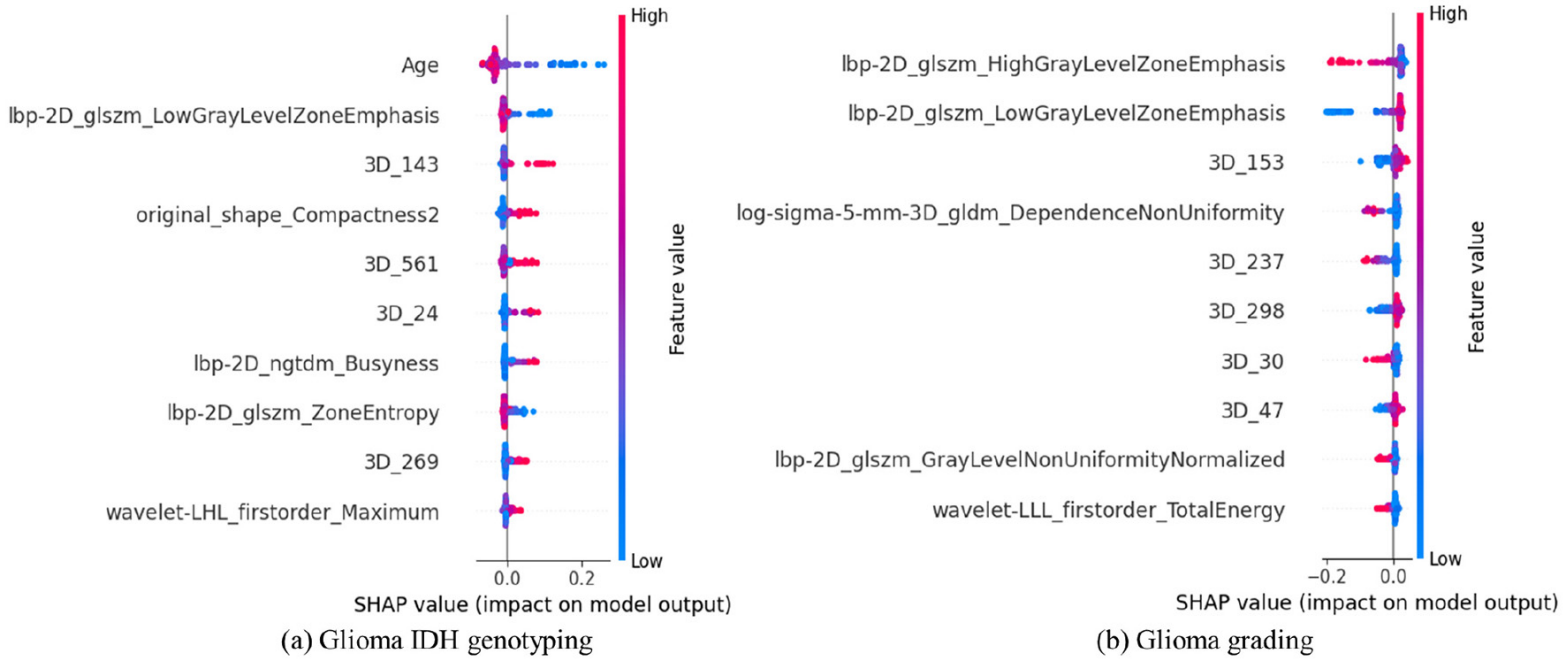
The top 10 most important 3D MRI, 1D HCR, and clinical features in MTSAM obtained by the SHAP method on the UCSF-PDGM dataset. The vertical axis represents the importance ranking of features, and the horizontal axis represents the SHAP value. Each point in the figure represents a sample, with colors reflecting the feature value from low (blue) to high (red). **(A)** Glioma IDH genotyping. **(B)** Glioma grading.

To explain the decision-making process of MTSAM, we pool the 3D MRI features obtained from the transformer block output in the fine-tuned SAM-Med3D across channels, name them sequentially, concatenate them with 1D HCR and clinical features, and perform SHAP analysis via linear regression on the UCSF-PDGM dataset. As shown in Figure 6a, we find that for IDH typing, age is the most important feature, as the larger its value, the more likely the glioma is predicted to be IDH wild type, which is consistent with the study by Reuss et al. (2015). In addition, the 143rd 3D MRI feature is important for glioma IDH typing, as the larger its value, the more likely the glioma is predicted to be IDH-mutant. As shown in Figure 6b, we find that for glioma grading, lbp-2D_glszm_HighGrayLevelZoneEmphasis is the most important feature, as the larger its value, the more likely the glioma is predicted to be a lower-grade glioma, which is consistent with the study by Mapelli et al. (2022). In contrast, the 153rd 3D MRI feature is also important for glioma grading: the larger its value, the more likely the glioma is to be higher grade.

## 4 Discussion

This study proposes a network, MTSAM, for simultaneous glioma IDH genotyping and grading using non-invasive data. Previous studies (Yu et al., 2024; Lim-Fat et al., 2022) mainly rely on invasive methods such as biopsies and tissue resections. In contrast, MTSAM utilizes non-invasive features, including multi-modal MRIs, which can effectively reduce patient discomfort and surgical risks. Specifically, MTSAM uses multi-view data to fine-tune the foundational model SAM-Med3D, aiming to accurately perform glioma IDH genotyping and grading. We validate MTSAM on the UCSF-PDGM and BraTS2020 datasets, and Table 2 shows that MTSAM outperforms other existing methods in glioma IDH genotyping and grading, demonstrating its potential in glioma diagnosis. Furthermore, Figure 4 visualizes the attention maps of MTSAM and other methods, validating MTSAM's potential for mining diagnosis-related features and demonstrating that leveraging the knowledge learned by medical foundational models from large-scale data may improve glioma diagnosis.

We validate the effectiveness of each module in MTSAM. Table 3 shows that when the MFEB is not used for T2-FLAIR mismatch feature extraction, the performance of MTSAM in glioma IDH genotyping and grading decreases, which reveals the potential of MFEB in mining T2-FLAIR mismatch features. Furthermore, Figure 5 demonstrates that, compared with the direct subtraction of T2 and FLAIR images, the T2-FLAIR mismatch features extracted by MFEB can more clearly highlight the mismatch phenomenon around the tumor region. Additionally, Table 3 indicates that using MVAdapter, fine-tuning the medical foundational model SAM-Med3D with multi-view information, can effectively improve glioma diagnostic performance, which may be attributed to the fact that multi-view information provides the model with more comprehensive and rich feature perspectives, thereby mitigating the information limitations of a single view. Moreover, Table 6 compares MVAdapter with other fine-tuning methods, validating the potential of MVAdapter in fine-tuning medical foundational models.

Figure 6 demonstrates the interpretability of MTSAM and visualizes the contributions of MRI features, HCR, and clinical features to glioma diagnosis. Specifically, we find that age is the most important feature for glioma IDH genotyping, as higher age is associated with a higher likelihood of IDH-wildtype gliomas. This is consistent with the fact that IDH-mutant gliomas often occur in young and middle-aged adults with active cell proliferation (Yamasaki, 2022; Weller et al., 2024). In contrast, the development of IDH-wildtype gliomas relies on the synergistic activation of multistep oncogenic pathways, such as EGFR amplification and TERT promoter mutation. These mutations require the accumulation of long-term DNA damage and a decline in cellular repair capacity as the basis. They mostly correspond to gliomas with higher malignancy, such as glioblastoma, and the risk of developing such tumors increases with age. For glioma grading, lbp-2D_glszm_HighGrayLevelZoneEmphasis is the most important feature, as higher values of this feature are associated with a higher likelihood of low-grade gliomas. This may be attributed to the fact that lbp-2D_glszm_HighGrayLevelZoneEmphasis is a feature designed to quantify large, continuous high-gray-level regions in images. Low-grade gliomas exhibit low malignancy, characterized by low cell density, minimal nuclear atypia, no obvious necrosis or angiogenesis, and more homogeneous tissue composition, which is reflected in MRI images as continuous, large-scale, and uniformly distributed high-gray-level tumor parenchyma regions, aligning with the quantification direction of lbp-2D_glszm_HighGrayLevelZoneEmphasis.

MTSAM exhibits considerable clinical value, with an inference time of only 0.82 s per sample, indicating high efficiency and promising it for rapidly providing glioma diagnostic results, thus offering auxiliary support for treatment decisions. Although MTSAM has achieved some progress in glioma IDH genotyping and grading, it still has several limitations. First, in real-world clinical settings, there is significant variation in scanner variability, imaging protocol heterogeneity, and patient population diversity, and the model has not yet been validated on larger datasets. Second, its potential for application in other tumor types remains unproven. Therefore, we plan to collect more data from real clinical scenarios and conduct validation across multiple tumor types to further verify the robustness and effectiveness of MTSAM.

## 5 Conclusion

In this study, we propose a multi-task network, MTSAM, based on the customized SAM-Med3D. MTSAM fuses multi-view information to fine-tune SAM-Med3D in order to extract deep features from multi-modal MRI and T2-FLAIR mismatch features for glioma IDH genotyping and grading. MTSAM achieves the best performance on the publicly available UCSF-PDGM and BraTS2020 datasets. Additionally, we use Grad-CAM to verify that MVAdapter fine-tunes SAM-Med3D by fusing multi-view information, enabling the network to effectively focus on the regions of gliomas that contain rich prognostic information.

## Data Availability

The original contributions presented in the study are included in the article/supplementary material, further inquiries can be directed to the corresponding author.
